# Comparing Discounting of Potentially Real Rewards and Losses by Means of Functional Magnetic Resonance Imaging

**DOI:** 10.3389/fnsys.2022.867202

**Published:** 2022-07-28

**Authors:** Mathieu Pinger, Janine Thome, Patrick Halli, Wolfgang H. Sommer, Georgia Koppe, Peter Kirsch

**Affiliations:** ^1^Department of Clinical Psychology, Central Institute of Mental Health, Medical Faculty Mannheim, University of Heidelberg, Heidelberg, Germany; ^2^Department of Theoretical Neuroscience, Central Institute of Mental Health, Medical Faculty Mannheim, University of Heidelberg, Heidelberg, Germany; ^3^Clinic for Psychiatry and Psychotherapy, Central Institute of Mental Health, Medical Faculty Mannheim, University of Heidelberg, Heidelberg, Germany; ^4^Institute of Psychopharmacology, Central Institute of Mental Health, Medical Faculty Mannheim, University of Heidelberg, Heidelberg, Germany; ^5^Bethanien Hospital for Psychiatry, Psychosomatics and Psychotherapy, Greifswald, Germany; ^6^Department of Psychology, University of Heidelberg, Heidelberg, Germany

**Keywords:** delay discounting, monetary incentive delay task, reward, aversion, fMRI

## Abstract

**Aim:**

Delay discounting (DD) has often been investigated in the context of decision making whereby individuals attribute decreasing value to rewards in the distant future. Less is known about DD in the context of negative consequences. The aim of this pilot study was to identify commonalities and differences between reward and loss discounting on the behavioral as well as the neural level by means of computational modeling and functional Magnetic Resonance Imaging (fMRI). We furthermore compared the neural activation between anticipation of rewards and losses.

**Method:**

We conducted a study combining an intertemporal choice task for potentially real rewards and losses (decision-making) with a monetary incentive/loss delay task (reward/loss anticipation). Thirty healthy participants (age 18-35, 14 female) completed the study. In each trial, participants had to choose between a smaller immediate loss/win and a larger loss/win at a fixed delay of two weeks. Task-related brain activation was measured with fMRI.

**Results:**

Hyperbolic discounting parameters of loss and reward conditions were correlated (*r* = 0.56). During decision-making, BOLD activation was observed in the parietal and prefrontal cortex, with no differences between reward and loss conditions. During reward and loss anticipation, dissociable activation was observed in the striatum, the anterior insula and the anterior cingulate cortex.

**Conclusion:**

We observed behavior concurrent with DD in both the reward and loss condition, with evidence for similar behavioral and neural patterns in the two conditions. Intertemporal decision-making recruited the fronto-parietal network, whilst reward and loss anticipation were related to activation in the salience network. The interpretation of these findings may be limited to short delays and small monetary outcomes.

## Introduction

Imagine having to choose between two monetary rewards: win €50 today or €100 in three months. What would you choose? Intertemporal choice tasks (ICTs) like these are used to assess *delay discounting* (DD), an aspect of decision-making whereby individuals attribute decreasing value to outcomes in the future (Gonçalves and Silva, 2015; Myerson et al., 2017; Yeh et al., 2020). This decrease over time is most commonly described with a hyperbolic discounting function (Myerson and Green, 1995). Inter-individual differences in the temporal discounting rates are associated with economic behavior, but also mental disorders like substance use disorder (Story et al., 2014; Cruz Rambaud et al., 2017; Amlung et al., 2019).

Compare the decision above with the following one: Lose €50 today or €100 in three months. What would you choose now? It is still an open question whether the cognitive decision-making process between these potential losses are the same as in the first example, only in the opposite direction. Therefore, comparing the behavioral and neural processes between temporal loss discounting (LD) and temporal reward discounting (RD) was the main goal of this study.

On a behavioral level, there appear to be inherent differences between LD and RD. Losses are discounted less steeply than rewards (Loewenstein and Prelec, 1992; Frederick et al., 2002; Estle et al., 2006; Mitchell and Wilson, 2010; Green et al., 2014). In other words, it appears as if losses in the distant future remain aversive. Not only are losses discounted less steeply, but also less frequently: around 20% of participants do not discount losses at all, another 20% exhibit reverse discounting, i.e., gravitating more towards immediate choices with increasing delay. In contrast, future rewards are discounted by more than 90% of participants (Gonçalves and Silva, 2015; Myerson et al., 2017; Yeh et al., 2020). Another commonly found difference between RD and LD is the lack of magnitude effects in LD: in RD, very large wins are less steeply discounted than small wins, whereas for LD it was found to be constant over a wide range of monetary outcomes (Johnson and Bickel, 2002; Mitchell and Wilson, 2010; Green et al., 2014; Yeh et al., 2020).

There also appear to be similarities between both processes. A stronger tendency to discount future losses has been associated with substance use disorder (Johnson et al., 2015; Cox et al., 2020). Different studies report vastly different correlation coefficients between discounting rates of losses and reward, ranging from strong to none at all (Chapman, 1996; Mitchell and Wilson, 2010; Halfmann et al., 2013; Myerson et al., 2017). To summarize, it remains unclear whether LD and RD represent the same cognitive process.

This uncertainty translates to the neural underpinnings of LD and RD, with only very few studies comparing neural correlates of RD and LD. There is considerable literature only for the neural correlates of RD: RD typically recruits brain areas which are related to executive control (frontal and parietal cortex, supplementary motor area), reward valuation (ventral striatum, amygdala, orbitofrontal cortex) and salience (insula, anterior cingulate cortex (Wesley and Bickel, 2014; Owens et al., 2019). Moreover, steeper RD is associated with altered activity in regions including the ventral striatum (VS), inferior frontal gyrus, anterior cingulate and medial PFC (Schüller et al., 2019).

Directly comparing LD and RD, Bickel et al. (2009) reported no significant differences between BOLD responses in RD and LD. Xu et al. (2009) reported a stronger BOLD response in the dorsolateral PFC and the posterior cingulate, the insula, the thalamus and the striatum during LD trials as compared with RD trials. Using dynamic causal modeling, the same group found distinct networks for gains and losses, whereby the valuation of losses and gains relies more on dorsolateral PFC and medial cortical regions, respectively (Zhang et al., 2018).

Whereas the neural underpinnings of reward- and aversion-related discounting have been rarely compared, a large number of studies have compared other processes that involve both rewarding and aversive consequences. One of these is reward and loss anticipation as measured by monetary incentive delay (MID) tasks (Kirsch et al., 2003; Knutson et al., 2005). Cognitive processes during this task include the valuation of possible outcomes and instrumental behavior to obtain a given outcome. For this task, recent meta-analyses have demonstrated comparable activation for trials involving losses and rewards, but also stronger activation in the ventral striatum during reward compared to loss anticipation (Dugré et al., 2018; Oldham et al., 2018). Like outcome anticipation, intertemporal decision-making usually includes an evaluation process and instrumental approach behavior (see also Scheres et al. (2013)). Therefore, differences in neural activation between loss and reward decision-making could be confounded with differences in the motivational value of the reward or loss as reflected in the activation during reward and loss anticipation (Algermissen et al., 2021). To this end, we developed a sequence of decision-making and outcome anticipation by combining intertemporal choice tasks with a MID task (Kirsch et al., 2003). This further allowed us to conduct a MID task with highly salient outcomes which have been chosen by the participants themselves.

In addition, behavioral modeling of discounting parameters allowed us to derive subjective values which we could associate with brain activation during the MID task. During decision-making, the subjective value of monetary wins is associated with stronger activation in the MPFC, the VS, the PCC, the ACC and other regions throughout the frontal and parietal cortex (Sripada et al., 2011; Schüller et al., 2019).

Most analyses were performed in an exploratory manner. We focused on investigating differences and correlations between behavior and neural activation during decision-making between rewards and losses. We further collected data on self-perceived impulsivity via the Barratt Impulsivity Scale-15 (Meule et al., 2011) to investigate its association with delay discounting. We preregistered seven hypotheses based on the results of an unpublished pilot study (for further information)^1^. Hypotheses included the presence of delay discounting in both reward and loss conditions, a replication of ventral striatal activation during reward anticipation, and more ventral striatal activity during reward anticipation than during loss anticipation. Furthermore, we hypothesized to see no correlation between behavioral parameters for LD and RD. Lastly, we expected an association between stronger RD and higher activation during the anticipation of immediate rewards (compared to delayed rewards), and an association between stronger LD and reduced prefrontal activation during decision-making.

## Methods

### Sample

We recruited 30 healthy participants from local universities via social media, public notices and registers of participants from earlier studies. Eligibility criteria included: absence of acute severe medical diseases, absence of acute psychiatric disorders, and MRI suitability. Eligibility criteria were assessed using a standardized telephone screening protocol. Eligible participants signed written informed consent prior to the study. There was no financial compensation, but participants could win money based on their responses during the experiment. On average, participants won €15.20 during the study.

The study was approved by the ethics committee of the Medical Faculty Mannheim, University of Heidelberg (2019-633N).

### Study Procedure

A standardized information sheet was used to explain the behavioral task before the MRI session. Participants were informed that they would be compensated based on their decisions in the behavioral task.

**FIGURE 1:**
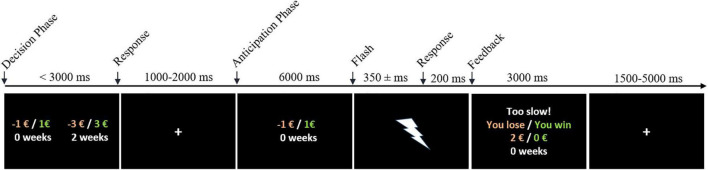
Experimental design. Arrows indicate onsets of GLM regressors. Participants had to choose between two losses (red) or two wins (green) within 3 seconds (*decision phase*). The chosen outcome was then cued for 6 seconds (*anticipation phase*), after which a short flash (50 ms) occurred. If participants pressed the button within an adaptive response window (starting with 300 ms), they received the outcome. If not, the win was reduced to €0 (reward condition) and losses were doubled (loss condition), as presented in the example.

Each participant completed the experiment in the fMRI scanner. The experiment consisted of two sessions of 32 trials each. Within each trial, the participant first had to choose between two monetary options (decision phase) and then respond quickly enough after an anticipation period to receive the chosen option (anticipation phase). The trial procedure is described below and illustrated in Figure 1.

The two sessions were identical in every aspect (amounts to choose from, order of stimuli) except for valence: participants had to choose between and anticipate monetary wins in one session (reward condition) and losses in the other session (loss condition). The order of the two sessions was counter-balanced across participants.

Decision Phase (Intertemporal Choice Task)*:* At the beginning of each trial, participants had to choose between a smaller immediate or a larger later amount of money to be received/lost in two weeks. The delay was always fixed at two weeks to allow for manageable payment. If no choice was made within 3 seconds, the trial was excluded for analysis. The phase was followed by a jittered 1-2 sec inter-stimulus interval.

The 32 trial options were calculated for eight fixed amounts for the immediate option (€1, €1.25, €1.50, €1.75, €2, €2.25, €2.50 and €2.75) and four ratios between the immediate and delayed options (0.2, 0.4, 0.6, 0.8). For example, the delayed options in the four trials offering €1 immediately were: €5, €2.5, €1.66, and €1.25.

Anticipation Phase (Incentive Delay Task): After making a choice, the chosen amount of money and the chosen delay (immediate or 2 weeks) were cued for 6 seconds. Subsequently, a short flash of 50 ms duration prompted the participant to respond as fast as possible by pressing a button to receive the chosen outcome. The threshold for a fast response was adaptive for each trial, targeting a 50% probability of success: starting with 300 ms, the required reaction time was increased/decreased by 5% after a slow/fast response. Then the feedback was presented for 1.5 seconds. In case of no or a too slow response, the chosen reward was replaced with €0, whereas chosen losses were doubled. Lastly, the jittered inter-trial interval of 1.5 to 5 seconds followed.

For each task, two trials were randomly selected and paid out. For the trials of the loss discounting experiment, participants were given a baseline balance of €8.20 for immediate choices and €10 for delayed choices, from which the selected loss was subtracted. An equal balance for both choices would have resulted in a trivial task where the smaller loss would constantly yield a larger win. We selected different balances so that choosing the delayed loss would result in a higher win in 50% of trials.

After the MRI session, participants were asked to fill out the Barratt Impulsivity Scale-15 (BIS-15, German Short Version; Meule et al. (2011)) and two open questions for each task: 1) *Did you have a strategy (if yes, please describe)?* 2) *Did you switch your preference for an immediate or delayed win/loss at a specific difference between the two amounts?* These questions were used to assess whether we successfully induced discounting of losses despite the possibility of winning money in both tasks.

Behavioral data extracted from logfiles included individual trial-wise choices and reaction times during the decision and anticipation phases.

### Behavioral Modeling

We inferred hyperbolic delay discounting models on the sequence of behavioral choices of each subject (Mazur, 1987; Davison and McCarthy, 1988), as commonly done in human research (Mazur, 1987; Davison and McCarthy, 1988; Kirby and Herrnstein, 1995; Myerson and Green, 1995; Johnson and Bickel, 2002; Ballard and Knutson, 2009; Bernhardt et al., 2019; Ahn et al., 2020; Croote et al., 2020). Since we have presented only two delays, robust estimation of discounting models is limited. Therefore, we did not run model comparisons between different discounting models and instead applied the commonly used hyperobic model to remain comparable to other studies. The model assumes that the internal (subjective) values *V* of a delayed choice *a_2_* decline hyperbolically over time, i.e., according to *V*(*a*_2_) = $\left(\frac{1}{1+\cdot \kappa^{l}D}\right)\,r_{2}$, where *r*_2_ represents the outcome of the delayed option, κ*^l^* is a free (discounting) parameter reflecting the individual tendency of discounting the delayed outcome in the reward or loss condition (indexed by *l*), and *D* represents the temporal delay. The value of the immediate choice *V*(*a*_1_) is simply given by the outcome *r*_1_ itself.

By connecting these values to behavioral choices in the task through a probabilistic process, we describe the probability *p* for choosing an action *a_i_* as $p\,\left(a_{i}\right)=\frac{e^{\frac{1}{\beta^{l}}\,V\,\left(a_{i}\right)}}{\sum_{j}e^{\frac{1}{\beta^{l}}\,V\,\left(a_{j}\right)}},$ where β*^l^* describes the individuals tendency to exploit or explore choices (separately for reward and loss conditions). Parameters were inferred via maximum likelihood estimation using constrained parameter optimization (with inbuilt MATLAB routines) and parameter constraints on β*^l^*∈ [0, 1], and κ*^l^*∈ [0, ∞).

Furthermore, the (negative) subjective values of chosen losses were transformed to absolute subjective values for easier interpretation (so that a higher absolute subjective value reflects a higher loss).

### Behavioral Data Analysis

To evaluate discounting behavior during the decision phase, we first counted the individual number of discounted choices by condition, that is, all immediate choices in the reward condition and all delayed choices in the loss condition. To test the hypothesis that the frequency of discounted choices increased as a function of a higher immediate/delayed ratio, we aggregated the data by obtaining the relative frequency of discounted choices for each participant, condition and ratio.

Based on these data, we set up a linear mixed model (LMM, Singmann and Kellen (2019)) to test for the effect of ratio (between the immediate and delayed options) and condition on the number of discounted choices. The LMM was chosen to take into account the hierarchical data structure and possible interaction effects. Here, the outcome variable was the relative frequency of discounted choices, with condition (i.e., reward/loss) and ratio between immediate and delayed amount (i.e., 0.2, 0.4, 0.6, 0.8) as fixed effect predictors. Furthermore, we added a per-participant random intercept, a random slope per participant for both fixed effects, and the correlation between the random effects, resulting in the following formula:

<>Relative _ frequency ∼ condition + ratio + (condition + ratio | subject)<>

Next, we tested whether the behavioral parameters κ and β from the hyperbolic model were significantly different or associated between conditions. To this end, paired t-tests and Pearson’s correlation coefficient were calculated. To rule out potential bias from non-converging behavioral models, these statistics were repeated excluding participants without choice variability in at least one condition. Lastly, correlations between the model parameters, the number of discounted choices, and BIS-15 scores were calculated.

To investigate other possible differences between loss and reward trials, we statistically compared reaction times during loss and reward trials, both for the decision phase and the anticipation phase. Here we also took into account the ratio between monetary options (decision phase) and the reward/loss magnitude (anticipation phase) as possible predictors of reaction time. To this end, we fit LMMs to the data of both phases.

For the decision phase, we set up a LMM with the reaction time during decision-making as outcome variable. Fixed effect predictors were condition (reward/loss) and ratio between immediate and delayed amount (i.e., 0.2; 0.4, 0.6, 0.8). Furthermore, we added a per-participant random intercept, a random slope per participant for both fixed effects, and the correlation between the random effects, resulting in the following formula:

<>Reaction_time ∼ condition + ratio + (condition + ratio | subject)<>

For the anticipation phase, we set up a LMM with the reaction time after the flash as an outcome variable. Fixed effect predictors were condition (reward/loss) and outcome magnitude, which we obtained from the subjective values derived from the hyperbolic model. A random intercept per participant was included, resulting in the formula:

<>Reaction_time ∼ condition + subjective_value + (1 | subject)<>

A significance threshold of p <.05 (two-sided) was used for all behavioral analyses. All behavioral analyses were performed using R (version 4.1.2). Linear mixed models were fit using the packages *lme4* (Bates et al., 2015). F-statistics and p-values for LMMs were estimated using the Satterthwaite method as implemented in the statistical R package *lmertest* (Kuznetsova et al., 2017).

### Brain Imaging

Functional imaging data were acquired using a 3 Tesla Siemens Magnetom Trio Scanner (Siemens Medical Systems, Erlangen, Germany) with a 32 channel head coil. Morphological brain data was assessed by high-resolution 3-dimensional T1-weighted anatomical images (MPRAGE) (repetition time (TR) = 2300 ms, eco time (TE) = 3.03 ms, flip angle = 9°, field of view (FOV) = 256 mm, 192 slices, slice thickness = 1.00 mm, voxel dimension = 1.0 × 1.0 × 1.0 mm, matrix size = 256 × 256).

The individuals blood oxygen level dependent (BOLD) signal was measured with two 9:36 min T2*-weighted echo-planar image (EPI) sequences with 285 volumes (TR = 2000 ms, TE = 30 ms, flip angle = 80°, FOV = 192 mm, 28 sagittal slices, slice thickness = 4.0 mm, 1 mm gap, voxel dimension = 3.00 x 3.00 x 4.00 mm, matrix size = 64 x 64). The behavioral tasks were presented using the Presentation software package (Version 21.1, Neurobehavioral Systems, Inc., Albany, CA, United States).

### Functional Magnetic Resonance Imaging Data Analysis

#### Preprocessing

We used SPM 12 (Wellcome Department of Cognitive Neurology, London, United Kingdom) implemented in MATLAB R2019a (MathWorks Inc., Sherborn, MA, United States) for preprocessing and analysis of functional images. The first four volumes of functional data were discarded. Preprocessing included normalization of the anatomical image to the SPM TPM template, and for the functional images slice-time correction, realignment to the mean image, co-registration to the anatomical image, spatial normalization to the SPM TPM template, rescaling to a resolution of 2 mm × 2 mm × 2 mm, and spatial smoothing with a 8x8x8 mm Gaussian kernel.

#### Modeling

For subject-specific first-level analyses, we set up three general linear models (GLMs). The reward and loss conditions were modeled as separate sessions within the GLMs described below. The respective regressors were the same for both sessions and are illustrated in Figure 1. Regressor onsets were convolved with the default SPM canonical hemodynamic response function. Six estimated movement parameters were included as regressors of non-interest in all models. The following GLMs were specified:

(1). A *phase-related GLM* was set up to compare task-related activation between conditions (i.e., loss/reward) and implicit baseline. Two phase related-regressors of interest were specified: the *decision phase* (onset of the decision phase modeled with the respective reaction time) and the *anticipation phase* (onset of the cue during the anticipation phase modeled with a fixed duration of 6 s). Trials in which the participants failed to choose an option during the decision phase were excluded from the regressors of interest by adding two dummy regressors of non-interests. To account for additional activation variance of no interest, we added several regressors of no interest, including the button press during the decision phase, the flash after the anticipation phase, the button press after the flash, and the feedback.
  The following contrasts were specified to detect activation related to the reward decision phase (RDec), the loss decision phase (LDec), reward anticipation (RA) and loss anticipation (LA):
  Decision Phase: RDec > Implicit Baseline; LDec > Implicit Baseline; RDec > LDec; LDec > RDec.
  Anticipation Phase: RA > Implicit Baseline; LA > Implicit Baseline; RA > LA; LA > RA.

(2). A *parametric decision-related GLM* was set up to assess changes in brain activation in response to trial difficulty, which was operationalized as the difference between the subjective values (SV) of the immediate and delayed options. Here, a smaller difference indicates that the immediate option and the discounted delayed option have a more similar subjective value, which is considered a more difficult decision. The subjective value difference (SV_Diff) was added as a parametric modulator for the decision phase.
  Following contrasts were specified in this model:
  Decision Phase: SV_Diff_*reward*_ > Baseline; SV_Diff_*loss*_ > Baseline.

(3). A *parametric anticipation-related GLM* was set up to characterize the association between phase-related activation during the anticipation phase and the internal value representation of the cued amount of money. For this purpose, the subjective value of the chosen option was added as a parametric modulator for the anticipation phase.
  Following contrasts were specified in this model:
  Anticipation Phase: SV_*reward*_ > Baseline; SV_*loss*_ > Baseline.

(4). A *choice-related GLM* was set up to compare choice-related activation within conditions. The regressors were identical with the first model, with the two phase-related regressors of interest (decision phase and anticipation phase) being split into four regressors based on the participant’s choice for the immediate or delayed option. Participants with less than 20% discounted choices (6 out of 32 trials) were excluded from respective contrasts.
  The following contrasts were specified in this model:
  Decision Phase: Immediate > Delayed, Delayed > Immediate (separately for reward and loss).
  Anticipation Phase: Immediate > Delayed, Delayed > Immediate (separately for reward and loss).

Linear contrast estimates were then entered into a second-level random effects model. One-sample *t*-tests were used to detect within-group activation. Inferences were conducted on the whole-brain level with a cluster-corrected significance threshold of *p* < 0.05 and a cluster-defining threshold of *p* < 0.001 uncorrected. We also conducted all contrasts at a family-wise peak voxel-corrected threshold of *p* < 0.05. Both cluster-corrected and peak voxel-corrected *p*-values are reported in the (Supplementary Tables 1, 2). The contrasts for the *decision phase* (task-related GLM) yielded very large clusters (> 60.000 voxels), therefore we only report regions which remained significant at the peak voxel-corrected threshold.

In order to test the hypotheses regarding ventral striatal activation during reward and loss anticipation, an a priori defined ROI analysis was conducted for two contrasts of the first model: “Reward > Implicit Baseline” (Hypothesis 2) and “Reward > Loss” (Hypothesis 3). The mask for the ROI analysis covering the bilateral nucleus accumbens was based on the automated anatomical atlas (AAL, Tzourio-Mazoyer et al. (2002)) and comprised a volume of 9506 mm^3^ (1189 voxels). Inference for ROI analyses was conducted with a significance threshold of *p* < 0.05 corrected for small volume.

To test for associations between individual discounting tendency and brain activation during the *decision phase*, model-derived discounting parameters (κ) were entered as covariates of interest in the second level models. Lastly, to test the hypothesis of stronger RD and higher activation during the anticipation of immediate rewards (compared to delayed rewards), the discounting parameter κ^*reward*^ was entered as a covariate of interest in the second level of the *choice-related* contrast Immediate > Delayed Reward Anticipation.

## Results

### Study Population

The study sample consisted of 30 undergraduate university students (mean age ± SD = 24 ± 3.46 years; range 19 to 33; 14 female) from different fields.

### Missing Data

MRI data from two participants had to be excluded due to an incidental finding and excessive head movement. Behavioral analyses are reported with N = 30, fMRI analyses with N = 28.

A total of 30 trials (1.56% of all trials) had to be excluded from further analyses due to no decision within 3s.

### Behavior

**FIGURE 2:**
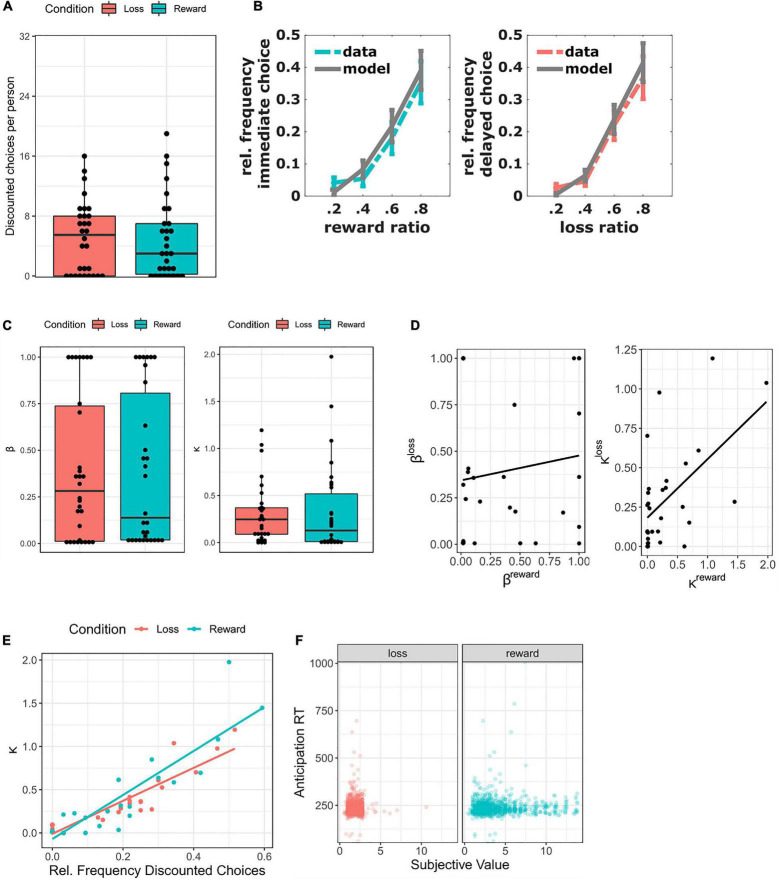
Behavioral results (*N* = 30). **(A)** Number of discounted choices per person (reward = immediate choices; loss = delayed choices). **(B)** Relative frequency of discounted choices per ratio between immediate and delayed options (means and standard errors of the mean). Gray: means and standard errors of the subject-wise hyperbolic model predictions. **(C)**: Distribution of κ and β values. **(D)**: Associations of κ and β between conditions. **(E)**: Association between κ parameters and relative frequency of discounted choices. **(F)**: Associations between reaction time during the anticipation phase and hyperbolic model-derived subjective values (note: absolute subjective values used for loss condition, “see Behavioral Modeling”).

For the decision phase, the number of discounted choices per condition is illustrated in Figure 2A. The average number of discounted choices (out of 32 trials) was 4.93 in the reward condition and 5.12 in the loss condition (Table 1), with a correlation of *r* = 0.47 (*p* = 0.01) between the two. Almost a third of all participants (8 in the loss condition, 9 in the reward condition) never chose the discounted option. In both conditions, the relative discounting frequency increased as the ratio between immediate and delayed options approximated 1, as illustrated by Figure 2B. This is further confirmed by the statistically significant effect of ratio (t(29.31) = 5.62, *p* < 0.001) in the LMM predicting relative discounting frequency by ratio and condition (Table 2). In contrast, the effect of condition was not significant (t(29.05 = 0.03, *p* = 0.79), indicating no difference between loss and reward trials with regard to number of discounted choices.

**TABLE 1:** Means, standard deviations, and correlations of behavioral data (*N* = 30).

Variable	*M*	*SD*	1	2	3	4	5	6	7	8	9
(1). κ (Reward)	0.33	0.48									
(2). κ (Loss)	0.30	0.32	0.56**								
			[0.25, 0.77]								
(3). β (Reward)	0.38	0.41	0.26	0.39*							
			[−0.11, 0.57]	[0.03, 0.66]							
(4). β (Loss)	0.39	0.39	0.28	0.18	0.14						
			[−0.09, 0.58]	[−0.20, 0.50]	[−0.23, 0.48]						
(5). Number of discounted choices (Reward)	4.93	5.40	0.90**	0.56**	0.30	0.20					
			[0.80, 0.95]	[0.25, 0.76]	[−0.07, 0.60]	[−0.17, 0.52]					
(6). Number of discounted choices (Loss)	5.13	4.79	0.42*	0.92**	0.29	0.33	0.47**				
			[0.07, 0.68]	[0.83, 0.96]	[−0.08, 0.59]	[−0.03, 0.62]	[0.14, 0.71]				
(7). BIS: Non-Planning	9.50	2.81	−0.15	0.03	−0.24	0.02	−0.20	0.13			
			[−0.49, 0.22]	[−0.34, 0.38]	[−0.55, 0.13]	[−0.34, 0.38]	[−0.52, 0.17]	[−0.25, 0.46]			
(8). BIS: Motor	10.83	2.29	−0.32	0.00	0.08	−0.04	−0.27	0.01	0.34		
			[−0.61, 0.04]	[−0.36, 0.36]	[−0.29, 0.42]	[−0.39, 0.33]	[−0.58, 0.10]	[−0.35, 0.37]	[−0.02, 0.62]		
(9). BIS: Attentional	8.73	1.64	0.11	0.01	-0.08	-0.25	0.05	-0.03	0.09	0.09	
			[−0.26, 0.45]	[−0.35, 0.37]	[−0.43, 0.29]	[−0.56, 0.12]	[−0.32, 0.40]	[−0.38, 0.34]	[−0.28, 0.44]	[−0.28, 0.44]	
(10). BIS: Total	29.07	4.66	−0.21	0.02	−0.14	−0.09	−0.24	0.07	0.80**	0.73**	0.45*
			[−0.53, 0.16]	[−0.34, 0.38]	[−0.47, 0.24]	[−0.44, 0.28]	[−0.55, 0.13]	[−0.30, 0.42]	[0.62, 0.90]	[0.50, 0.86]	[0.11, 0.70]

M and SD are used to represent mean and standard deviation, respectively. BIS = Barrat Impulsivity Scale. * indicates p < .05. ** indicates p < .01.

**TABLE 2:** Linear Mixed Model for the effect of condition (Loss = 0, Reward = 1) and ratio between immediate and delayed options (0.2, 0.4, 0.6, 0.8) on relative frequency of discounted choices during the decision phase (*N* = 30).

Level	Effect	Estimate	SE	t	*df*	P	5% CI	95% CI
Group	Intercept	–0.12	0.03	–3.43	35.36	< 0.01	–0.19	–0.05
Group	Condition	–0.01	0.03	–0.27	29.05	0.79	–0.07	0.05
Group	Ratio	0.57	0.10	5.62	29.31	< 0.01	0.36	0.77
Subject	SD (Intercept)	0.13						
Subject	Intercept*Condition	–0.40						
Subject	Intercept*Ratio	–0.92						
Subject	SD (Condition)	0.13						
Subject	Condition*Ratio	0.02						
Subject	SD (Ratio)	0.50						
Residual	SD	0.14						

*Estimates are in relative frequencies (0 to 1).*

The descriptive statistics for the behavioral model parameters κ and β are presented in Table 1 (see also Figure 2C). Paired t-tests revealed that discounting parameters κ (t(29) = −0.31, *p* = 0.76) and choice parameters β (t(29) = 0.16, *p* = 0.88) did not differ significantly between conditions. Excluding participants showing no behavioral variability yielded the same results (all *p* > 0.44). Instead, model-derived discounting parameters κ (*r* = 0.56, *p* < 0.01), but not choice parameters β (*r* = 0.14, *p* = 0.46), were significantly correlated between conditions (Table 1 and Figure 2D). The correlation between discounting parameters κ did not remain significant after excluding participants without behavioral variability in at least one of the two conditions (*r* = 0.43, *p* = 0.09, *n* = 17).

Correlations between the behavioral model parameters and number of discounted choices are presented in Table 1. Discounting parameters κbased on the hyperbolic model were highly correlated with the number of discounted choices, both for the loss (*r* = 0.92, *p* < 0.001) and the reward condition (*r* = 0.90; *p* < 0.001; Figure 2E). The correlation remained constant after removing data from participants without behavioral variability (reward: *r* = 0.89; loss: *r* = 0.92, both *p* < 0.001). Taken together, the hyperbolic discounting parameters were a good indicator of observed discounting behavior.

No behavioral index of discounting behavior showed a significant association with any of the subscales nor the total scale of the BIS-15 (Table 1), indicating no linear relationship between self-rated impulsivity and observed discounting behavior.

Lastly, there was no effect of condition (reward/loss) on the reaction times in the decision phase and the anticipation phase. For the decision phase, the LMM revealed a statistically significant effect of ratio (t(28.67) = 4.06, *p* < 0.001)), but not condition (t(28.99) = −0.97, *p* = 0.34; Table 3), indicating slower decision-making in trials with higher ratio between the immediate and delayed option.

**TABLE 3:** Linear Mixed Model for the effect of condition (Loss = 0, Reward = 1) and ratio between immediate and delayed options (0.2, 0.4, 0.6, 0.8) on reaction times during the decision phase (*N* = 30).

Level	Effect	Estimate	SE	t	*df*	P	5% CI	95% CI
Fixed	Intercept	1, 043.17	35.58	29.32	28.90	< 0.01	970.40	1, 115.95
Fixed	Condition	–34.82	35.81	–0.97	26.99	0.34	–108.07	38.42
Fixed	Ratio	241.67	59.57	4.06	28.68	< 0.01	119.77	363.57
Subject	SD (Intercept)	167.13						
Subject	Intercept*Condition	–0.52						
Subject	Intercept*Ratio	0.59						
Subject	SD (Condition)	181.01						
Subject	Condition*Ratio	–0.02						
Subject	SD (Ratio)	279.04						
Residual	SD	299.79						

*Estimates are in milliseconds.*

For the anticipation phase, the LMM revealed no significant effects of condition (t(1852.64) = 0.01, *p* = 0.99) or subjective value (t(1856.64) = 0.29, *p* = 0.77; Table 4). The reaction time after the flash was therefore unrelated to valence or magnitude, as illustrated by Figure 2F.

**TABLE 4:** Linear Mixed Model for the effect of condition (Loss = 0, Reward = 1) and subjective value of outcome on reaction times during the anticipation phase. Subjective values of the chosen option were derived from the hyperbolic model (“see Behavioral Modeling”) (*N* = 30).

Level	Effect	Estimate	SE	t	*Df*	p	5% CI	95% CI
Fixed	Intercept	239.72	3.91	61.32	39.40	0.00	231.82	247.63
Fixed	Condition	0.05	3.81	0.01	1, 853.64	0.99	–7.42	7.53
Fixed	Subjective Value	0.16	0.53	0.29	1, 857.64	0.77	–0.89	1.20
Subject	SD (Intercept)	18.91						
Residual	SD	46.86						

*Estimates are in milliseconds.*

### Functional Magnetic Resonance Imaging

#### Decision Phase

##### Reward Condition

**FIGURE 3:**
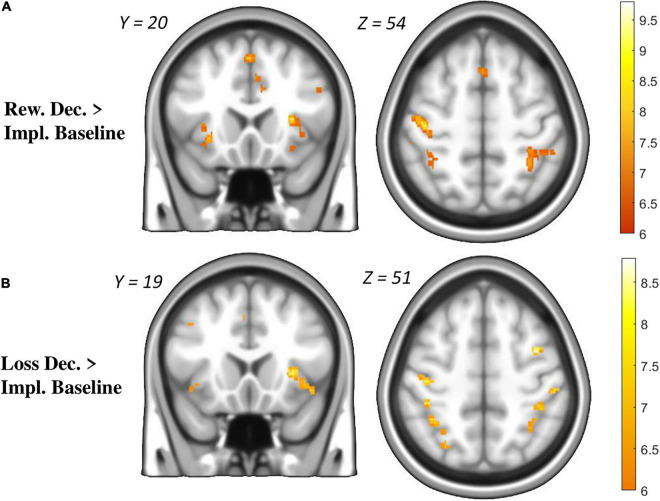
Brain activation during the decision phase (intertemporal choice task). **(A)**: Reward Decision Phase > Implicit Baseline **(B)**: Loss Decision Phase > Implicit Baseline. Results displayed at *p* < 0.5 FWE-corrected for multiple testing.

The *phase-related GLM* contrast “RDec > Implicit Baseline” revealed clusters of activation in the visual cortex, the cerebellum, the anterior insula, the operculum, the primary and supplementary motor areas, the superior and posterior parietal cortex, and the anterior cingulate cortex (see Figure 3A and Supplementary Table 1).

In no brain region was task-related activation significantly correlated to the individual discounting parameter ^κ*reward*^ (*phase-related GLM* contrast ‘RDec > Implicit Baseline) * ^κ*reward*^ ‘). This result remained unchanged after excluding participants without behavioral variability (remaining *n* = 21).

The *choice-related GLM* contrasts “Immediate > Delayed Choices” and vice versa revealed no significant clusters of brain activation. However, due to the low behavioral variance, data of only 12 participants were included in this contrast.

The *parametric modulation* of RDec with the subjective value difference between immediate and delayed options revealed no significant clusters of activation.

##### Loss Condition

The *phase-related GLM* contrast “LDec> Implicit Baseline” revealed clusters of activation in the visual cortex, the cerebellum, the anterior insula, the operculum, the primary and supplementary motor areas, the superior and posterior parietal cortex, and the anterior cingulate cortex (see Figure 3B and Supplementary Table 1).

No brain region showed activation in significant relation with the individual discounting parameter ^κ*loss*^ (*phase-related GLM* contrast ‘LDec > Implicit Baseline) * ^κ*loss*^ ‘). This result remained unchanged after excluding participants without behavioral variability (remaining *n* = 22).

The *choice-related GLM* contrasts “Immediate > Delayed Choices” and vice versa revealed no significant clusters of brain activation. However, due to the low behavioral variance, data of only 15 participants were included in this contrast.

The *parametric modulation* of LDec with the subjective value difference between immediate and delayed options revealed no significant clusters of activation.

##### Reward Condition vs. Loss Condition

There were no significant clusters in the *phase-related GLM* contrasts “LDec > RDec” and vice versa. An exploratory contrast combining loss and reward (‘LDec + RDec > Baseline’) revealed activation throughout the same regions as during the individual conditions (Supplementary Figure 1 and Supplementary Table 3).

#### Anticipation Phase

##### Reward Anticipation

**FIGURE 4:**
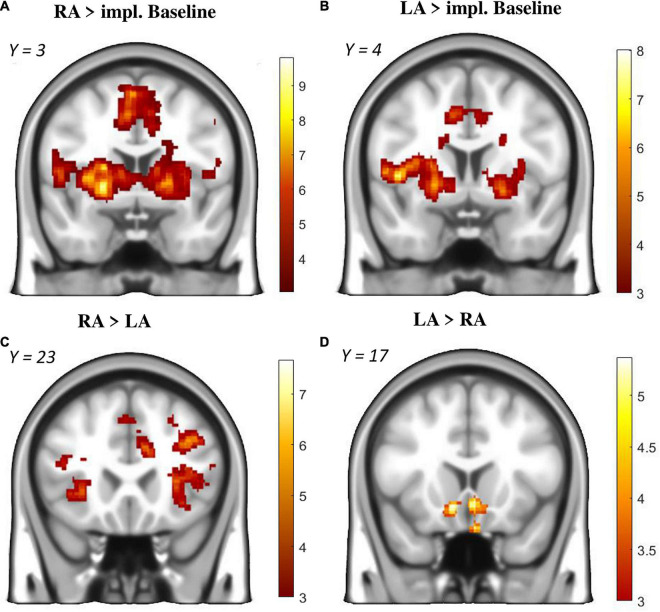
Brain activation during the anticipation phase (monetary incentive delay task). **(A)**: Reward Anticipation (RA) > Implicit Baseline. **(B)**: Loss Anticipation (LA) > Implicit Baseline. **(C)**: RA > LA. **(D)**: LA > RA. Only voxels from significant clusters (cluster-size *p* < 0.05 corrected for multiple testing and *p* < 0.001 as cluster-defining threshold) are displayed.

In the *phase-related GLM* contrast “RA > Implicit Baseline,” significant activation was present in the anterior insula, anterior cingulate cortex, putamen, pallidum, operculum, cerebellum, thalamus as well as primary and supplementary motor areas (see Figure 4A and Supplementary Table 2). Furthermore, the a priori ROI analysis revealed significant activation in the ventral striatum (Supplementary Table 2).

**FIGURE 5:**
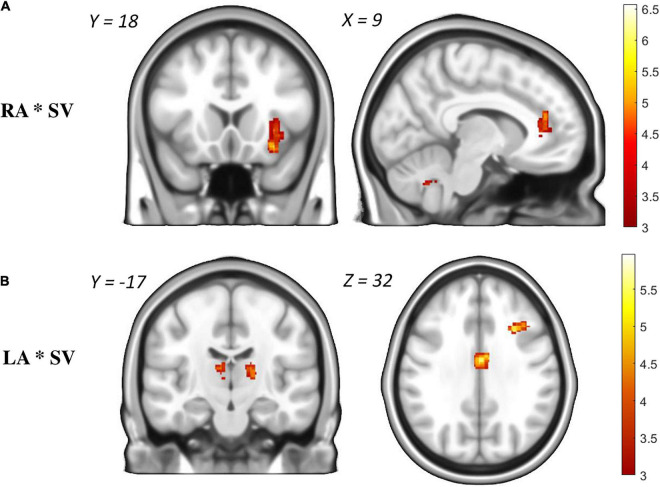
Parametric modulation of brain activation during the anticipation phase with hyperbolic model-derived subjective values (“see Behavioral Modeling”). **(A)**: Brain regions that show more activation during Reward Anticipation (RA) when subjectively higher rewards could be won. **(B)**: Brain regions that show more activation during Loss Anticipation (LA) when subjectively higher losses could be avoided. Only voxels from significant clusters (cluster-size *p* < 0.05 corrected for multiple testing and *p* < 0.001 as cluster-defining threshold) are displayed.

The *parametric modulation* of RA with subjective value revealed three clusters in the anterior insula + striatum, the cerebellum and the anterior cingulate cortex (Figure 5A and Supplementary Table 2). In other words, the chance of winning higher rewards was associated with more activity in salience-related regions.

The *choice-related GLM* contrasts “Immediate > Delayed Choices” and vice versa revealed no significant clusters of brain activation. However, due to the low behavioral variance, data of only 12 participants could be included in this contrast.

The *choice-related* brain-behavior correlation ‘(Immediate > Delayed) * ^κ*reward*^ revealed no significant clusters of activation.

##### Loss Anticipation

The *phase-related GLM* contrast “LA > Implicit Baseline” revealed significant clusters in the anterior insula, anterior cingulate cortex, putamen, pallidum, operculum, cerebellum, visual cortex as well as and primary and supplementary motor areas (Supplementary Table 2 and Figure 4B).

The *parametric modulation* of LA with subjective value revealed five clusters associated with the chance of preventing higher losses: the prefrontal cortex, middle cingulate cortex, thalamus and precuneus (Supplementary Table 2 and Figure 5B).

The *choice-related GLM* contrasts “Immediate > Delayed Choices” and vice versa revealed no significant clusters of brain activation. However, due to the low behavioral variance, data of only 15 participants reward could be included in this contrast.

##### Reward Anticipation vs. Loss Anticipation

The *phase-related GLM* contrast “RA >LA” revealed significantly more activation during RA throughout the prefrontal and parietal cortex, anterior insula, putamen, anterior cingulate cortex and motor areas (Supplementary Table 2 and Figure 4C). The opposite contrast “LA > RA” revealed significantly more activation during LA in the ventral striatum (Supplementary Table 2 and Figure 4D).

## Discussion

### General Discussion

In this study, we aimed to investigate the differences and/or associations between temporal discounting of losses and rewards on a behavioral and neural level. We combined an intertemporal choice task with a monetary incentive delay task in an fMRI experiment. That is, participants had to choose between two potentially real losses or rewards in the *decision phase* of each trial, and respond quickly to receive the chosen option in the *anticipation phase* of each trial. Individual discounting parameters were estimated based on the hyperbolic discounting model. Based on these parameters, we were able to obtain subjective values for the chosen rewards and losses and used these as parametric modulators in the fMRI models.

Statistical analyses focused on the exploratory comparison of the behavior and neural activation during the loss and reward conditions. During the decision phase of the task, we observed correlated LD and RD behavior and no neural differences between the loss and reward condition. During the anticipation phase, the ventral striatum was more strongly activated during the loss condition, whereas several regions including ACC and anterior insula were more strongly activated during the reward condition. Higher subjective losses and rewards were found to be associated with stronger activation in several regions during the anticipation phase.

Our a priori-defined analyses regarding correlations between neural activation and individual discounting parameters did not yield any significant finding. As discussed further below, this might be explained by the overall low rate of discounting behavior.

### Comparing Discounting of Rewards and Losses

We observed discounting of both losses (LD) and rewards (RD): participants gravitated more towards the discounted option in trials where the immediate and delayed option were more similar, i.e., where the ratio between options was closer to 1 (Table 2). The general discounting frequency in our experiment was low, which is why key analyses were repeated excluding non-discounters. However, at least for the loss task, the discounting rates seem in line with prior literature reporting about 30% non-discounters (Mitchell and Wilson, 2010; Myerson et al., 2017; Yeh et al., 2020; Thome et al., 2022).

In general, the observed data in both conditions matched the pattern predicted by the hyperbolic model (Figure 2B). Note that the hyperbolic model was chosen based on the literature (“see Behavioral Modeling”). With only one delay, it could not be compared to other models like the exponential or hyperboloid function. However, hyperbolic κ parameters were strongly correlated with the number of discounted choices in both reward (*r* = 0.90) and loss (*r* = 0.92) conditions, a result which remained unchanged after excluding non-discounting participants. These findings support our hypothesis of some form of discounting behavior in both conditions.

Contrary to our expectations, we found very similar behavior during loss and reward trials. The condition had no significant effect on the number of discounted trials (Table 2). Further, loss and reward behavior was highly correlated, as reflected by the correlation between conditions regarding number of discounted choices (*r* = 0.47, Table 1) and κ values (*r* = 0.56, Figure 2D). The strong correlation between κ values did not remain significant after excluding non-discounters, yet the effect size remained comparable (*r* = 0.46). Power analyses revealed that our full sample of *N* = 30 yielded 96% power to detect the strong correlation of *r* = 0.55 reported for middle-aged adults by Halfmann et al. (2013), but only 73% power to replicate the moderate correlation of *r* = 0.39 reported by Mitchell and Wilson (2010). Therefore, the lack of significance after reduction of the sample size can most likely be attributed to a lack of power.

Like the number of discounted choices, the behavioral model parameters κ and β did not significantly differ between conditions. However, prior studies (Chapman, 1996; Engelmann et al., 2013) reported differences between LD and RD only for large but not for small delays, matching our data which is only based on small delays. In addition, post-hoc power analyses revealed only 48% power to find a small difference of *d* = 0.3 between RD and LD in our sample. Consequently, our finding of no behavioral difference should not be generalized to large delays and small effects.

Another similarity between conditions was found in reaction times during the decision phase, which were highly variable, but not related with the reward/loss condition (Tables 3, 4). Together, this suggests commonalities between cognitive processes involved in reward and loss discounting, if only for short delays and small monetary outcomes.

### Successful Induction of Loss Perception

The implementation of real losses is not a straightforward enterprise if participants start with upfront money that can be won. Using one balance for both immediate and delayed losses would motivate participants to exploit the immediate loss to receive more money at the end. Here we tested a system with different balances for immediate and delayed losses: In this study, two random choices per condition were selected and paid out. Chosen losses were subtracted from a fixed balance if the reaction time during the anticipation phase task was fast enough and doubled if the reaction time was too slow. However, this means that participants could actually win money in the loss condition. By choosing different balances for the immediate (€8.20) and delayed loss (€10), we tried to prevent an obvious winning strategy. However, theoretically participants could always opt for the maximum win if they followed an optimal strategy. This means that we could not guarantee that the loss ICT actually induced a perception of losing money, rather than of potentially winning more money.

To evaluate our success in inducing a perception of loss (and consequently loss discounting), participants answered open questions about their strategy after completing all measurements. In the reward condition, 24 participants stated an overall strategy of always choosing the higher win, whereas 12 participants explicitly named a variable strategy based on time and reward ratio (discounting behavior). In the loss condition, 19 participants stated an overall strategy of always choosing the smaller loss, with 11 participants naming variable strategies based on time and reward ratio. Here, a variable strategy could be the result of both discounting and a win-oriented strategy. However, only one participant explicitly described choosing the loss that resulted in the larger win. Notably, 19 participants explicitly used the word “loss” when talking about their strategy, indicating a perception of actual losses, rather than absolute wins. Few studies have investigated LD in the fMRI, and to our knowledge, none have used real losses. This is understandable, given the obvious ethical problems of inflicting monetary losses on participants. Here we tried to mask the “optimal” choice by using different balances from which immediate and delayed losses were subtracted. This enabled us to follow up the intertemporal choice task by means of an incentive delay task with real losses. Taken together, the quantitative and qualitative results clearly indicate that the behavioral variance in the loss condition was indeed due to LD and not win-orientation.

### Brain Activation During Intertemporal Decision-Making

During the decision phase, a pattern of regions related to networks of salience (e.g., anterior insula and cingulate cortex), decision-making (e.g., parietal and frontal cortex) and motor control (e.g., precentral gyrus and SMA) was observed in both conditions (Figure 3 and Supplementary Figure 1). This activation pattern closely matches the overlap of three different discounting tasks against baseline reported by Koffarnus et al. (2017). In line with the behavioral results, we observed no significant differences between the loss and reward conditions. Furthermore, we found no brain-behavior correlation with the model-derived κ parameters. Again, the low behavioral variability observed throughout the experiment may have impeded the statistical comparison of neural discounting processes. Indeed, the few studies comparing neural correlates of LD and RD have reported inconsistent results (Bickel et al., 2009; Xu et al., 2009) which might suggest only subtle differences in neural activity during LD and RD.

We did not find any evidence for a modulation of brain activation by the difference between the two subjective values presented in each trial. Very difficult trials (i.e., trials close to the indifference point, therefore small difference) have been shown to elicit more activation in the ACC and dlPFC, than very easy trials (Monterosso et al., 2007; Koffarnus et al., 2017). Again, this result may be best explained by the small monetary outcomes and low behavioral variability, yielding only few trials with very hard and very easy choices.

### Delay Discounting and Impulsivity

No subscale or total score of the BIS-15 was significantly associated with discounting behavior. Excessive delay discounting is associated with “impulsive” behavior such as obesity and substance use disorders (Story et al., 2014). However, the term impulsivity is multi-faceted and contains different constructs with inconsistent associations (Moreira and Barbosa, 2019). Stahl et al. (2014) found no association between delay discounting and other measures of impulsivity and argue that “impulsivity” is an umbrella term of limited usability. The BIS-15 is a common self-rating instrument with weak associations to discounting at best (Reynolds et al., 2006; de Wit et al., 2007; Mobini et al., 2007). In conclusion, rather than reflecting impulsivity, discounting might better be understood as a construct on its own (Odum, 2011).

### Brain Activity During the Anticipation Phase

The observed activity during the anticipation phase in both conditions (Figure 4) matches the very typical pattern associated with motivational salience and monetary incentive delay tasks (Kirsch et al., 2003; Bjork et al., 2012; Oldham et al., 2018). The anterior insula and cingulate cortex are thought to modulate attention and goal-directed behavior towards context-relevant stimuli. Indeed, activity in these two regions was furthermore associated with subjective value, indicating a neuronal reflection of salience increasing with the amount of money that can be won or lost. In contrast, a study by Diekhof et al. (2012) found increased striatal response during the MID in response to an increased subjective value of the presented outcome. In our study, ventral striatal activation was surprisingly limited during reward anticipation, as reflected by the small cluster which only remained significant in the ROI analysis (Supplementary Table 2). A reason for this might be our overall experimental design with two tasks that are known to activate the ventral striatum (Kirsch et al., 2003; Schüller et al., 2019). In addition to this, the anticipation task lacked an explicit control condition, leaving only the implicit baseline contrast with possibly low residual variance.

### Comparing Reward and Loss Anticipation

Comparing baseline contrasts side by side, activity during loss anticipation was focused around the same salience hubs as during reward anticipation (Figure 4). This is in line with recent meta-analyses suggesting a valence-independent processing of motivational salience (Dugré et al., 2018; Oldham et al., 2018). However, a direct contrast of reward anticipation >loss anticipation and vice versa revealed differentiated activity during the two conditions (Figures 4C, D). The aforementioned salience regions and several clusters throughout the cortex were significantly more activated during reward trials, whilst loss trials were associated with more activation in the ventral striatum. The former effect is less surprising if we take into account the average subjective value: participants chose smaller losses and larger rewards (see also Figure 2F). Therefore, reward trials (with an average win of €4.02) were possibly more salient than loss trials (with an average loss of €1.89), reflected by more activity on corresponding brain networks. Indeed, brain regions involved in evaluating the motivational relevance of states have been theorized to act as valence-independent salience networks (Oldham et al., 2018).

Though plausible, this cannot explain the increased ventral striatal response during loss anticipation. In fact, we were expecting the opposite, as a recent meta-analysis reported more ventral striatal activity during reward anticipation (Oldham et al., 2018). However, given that the incentive delay task allows the participants to prevent an anticipated loss, increased activation in a core region of motivational processing might reflect a higher motivational value of the prevention of a potential loss compared to a potential win. Such a response pattern would be predicted by prospect theory (Kahneman and Tversky, 1979) in the context of loss aversion.

### Limitations

Our study used potentially real rewards and losses, trading external validity against a low monetary range and no variation in the delay, due to practical considerations. This combination probably resulted in a very low discounting rate, and hence statistical power. Moreover, generalization to large delays and monetary outcomes is limited. For most participants, the calculation of discounting parameters relied on few trials, limiting the reliability of the obtained parameters. Importantly, the lack of different delays made it impossible to compare different discounting models, e.g., the hyperbolical and the hyperboloid function.

Although we demonstrate a successful induction of loss discounting using real outcomes, this could possibly introduce a bias of upfront money, which might increase the validity of the monetary domain and hence reduce discounting (Jiang et al., 2016). Another possible bias comes from the payment procedure, where chosen losses were doubled if the anticipation time was too short. This unproportionally increased the potential loss associated with the (larger) delayed option.

Lastly, some methodological issues arise from our combination of an ICT with a MID. The contingency of the ICT outcome on performance in the MID induced ambiguity, i.e., an implicit and changing probability of ∼50% to not receive the chosen reward or even receive a higher loss. This may have biased participants to prefer a smaller loss. In addition, ambiguity may have influenced neural processes during the ICT, limiting the comparison to neuroimaging studies investigating pure delay discounting or risky decision-making (Ikink et al., 2019; Ortiz-Teran et al., 2019). Another limitation is that the behavioral paradigm was not designed to directly contrast brain activation during the decision phase and the anticipation phase. Therefore, the separation of decision-making and instrumental behavior is only conceptual, but cannot be backed up by more intricate statistical analyses.

### Future Research

A proper comparison of loss and reward discounting requires an adaptation of the paradigm with respect to inter- and intra-individual differences. Delay discounting is a decision-making process assumed to involve a valuation of options. Comparing subjective valuation requires comparable subjective values. This means taking into account global differences between RD and LD (e.g., magnitude effect, loss aversion) and individual discounting behavior to create intertemporal decisions with comparable subjective value. To this end, we recently developed a model-based framework to evoke predicted responses in RD and LD (Thome et al., 2022). As a next step, we plan to apply this adaptive task in the fMRI to allow for more fine-grained analysis of the neural differences between RD and LD.

## Conclusion

We found similar behavior in intertemporal choice tasks involving potentially real losses and rewards. Whilst the overall discounting rate was low, losses were discounted as frequently as rewards. There was a considerable correlation (*r* = 0.56) between hyperbolic discounting parameters κ during loss and reward discounting. In line with this finding, brain activation during reward- and loss-related decision-making were not significantly different from another. In contrast to that, reward anticipation recruited more salience-related brain regions like the anterior insula and the ACC, with the exception of more ventral striatal activation during loss anticipation. In line with prior research we demonstrate that brain activation in salience-related regions during reward and loss anticipation was associated with model-derived subjective values. Taken together, the general results of our study seem to support the account that LD and RD rely on similar or at least overlapping cognitive and neural processes. However, this similarity is yet to be demonstrated for an extensive range of delays and monetary outcomes.

## Data Availability Statement

The behavioral datasets presented in this study can be found in online repositories. The names of the repository/repositories and accession number(s) can be found below: https://osf.io/kmer3/. The fMRI datasets generated and analyzed during the current study are available from the corresponding author on reasonable request.

## Ethics Statement

The studies involving human participants were reviewed and approved by Ethics Committee of the Medical Faculty Mannheim, University of Heidelberg (2019-633N). The patients/participants provided their written informed consent to participate in this study.

## Author Contributions

GK, PK, and WS conceptualized the study. MP and PH collected the data. MP, JT, and GK performed the statistical analyses. MP wrote the manuscript. All authors contributed to conception and the design of the study, read, revised, and approved the submitted manuscript.

## Conflict of Interest

The authors declare that the research was conducted in the absence of any commercial or financial relationships that could be construed as a potential conflict of interest.

## Publisher’s Note

All claims expressed in this article are solely those of the authors and do not necessarily represent those of their affiliated organizations, or those of the publisher, the editors and the reviewers. Any product that may be evaluated in this article, or claim that may be made by its manufacturer, is not guaranteed or endorsed by the publisher.

## Footnotes

1
https://osf.io/cj35t
