# Supplementary figures and images for: Comparing Discounting of Potentially Real Rewards and Losses by Means of Functional Magnetic Resonance Imaging
Source: Front Syst Neurosci. 2022 Jul 28;16:867202. doi: 10.3389/fnsys.2022.867202 (PMC9365957; doi:10.3389/fnsys.2022.867202)

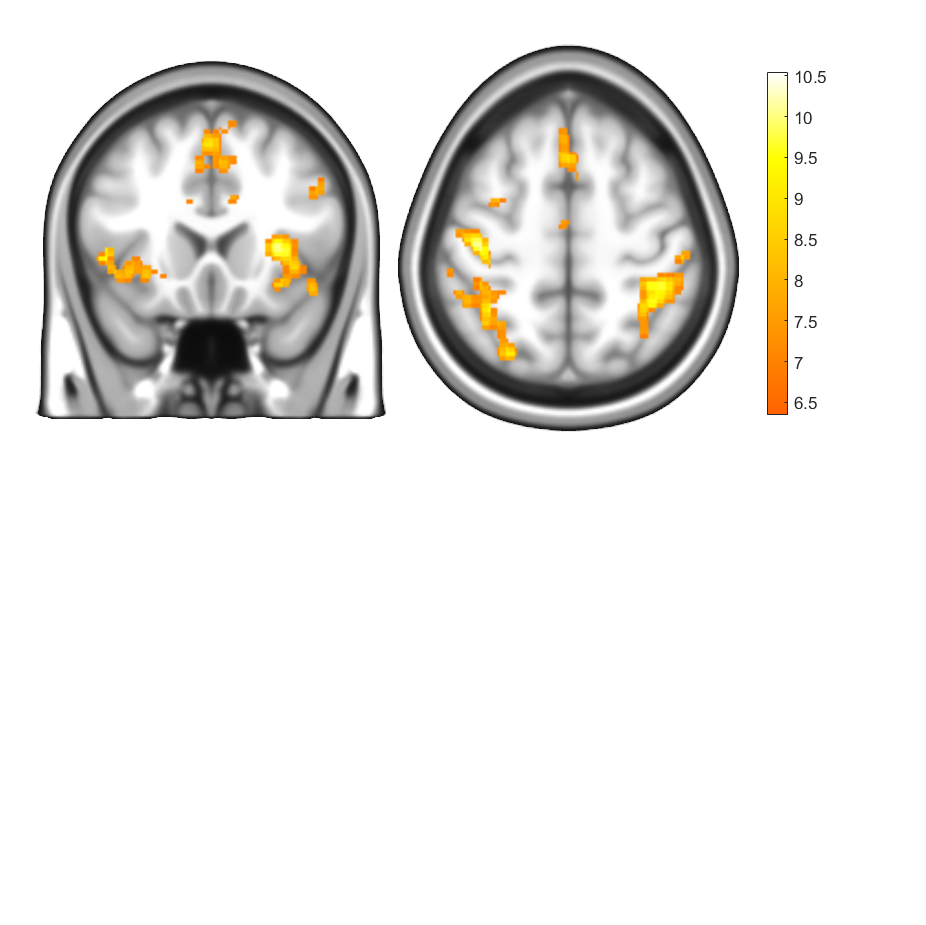

Supplement: Supplementary file 1 [file Image_1.TIF]
